# Correction: A novel G·G·T non-conventional intramolecular triplex formed by the double repeat sequence of *Chlamydomonas* telomeric DNA

**DOI:** 10.1039/d2ra90059a

**Published:** 2022-06-09

**Authors:** Aparna Bansal, Priyanka Phogat, Shrikant Kukreti

**Affiliations:** Nucleic Acids Research Lab, Department of Chemistry, University of Delhi (North Campus) Delhi 110007 India skukreti@chemistry.du.ac.in shrikant.kukreti6@gmail.com; Department of Chemistry, Hansraj College, University of Delhi (North Campus) Delhi 110007 India

## Abstract

Correction for ‘A novel G·G·T non-conventional intramolecular triplex formed by the double repeat sequence of *Chlamydomonas* telomeric DNA’ by Aparna Bansal *et al.*, *RSC Adv.*, 2022, **12**, 15918–15924, https://doi.org/10.1039/D2RA00861K.

The authors regret that an incorrect version of Fig. 6 was included in the original article. The correct version of Fig. 6 is presented below.

**Fig. 6:**
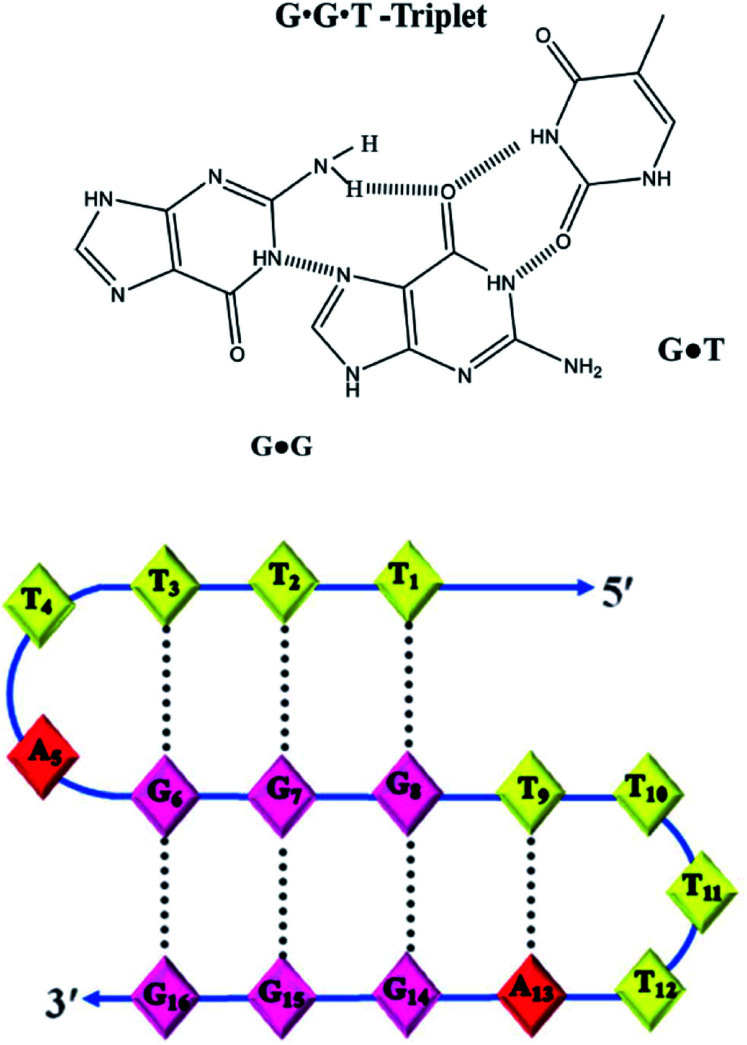
Proposed model of the non-conventional triplex comprising G·G·T triplets formed by Chlm2.

The Royal Society of Chemistry apologises for these errors and any consequent inconvenience to authors and readers.

## Supplementary Material

